# Editorial: Searching for causes of infertility: from pathophysiologic mechanisms to therapeutic strategies

**DOI:** 10.3389/fgene.2024.1432026

**Published:** 2024-06-26

**Authors:** Marzena Kamieniczna, Marta Olszewska, Agnieszka Malcher, Tomasz Stokowy, Sezgin Gunes

**Affiliations:** ^1^ Institute of Human Genetics, Polish Academy of Sciences, Poznan, Poland; ^2^ IT Division, University of Bergen, Bergen, Norway; ^3^ Faculty of Medicine, Department of Medical Biology, Ondokuz Mayis University, Samsun, Türkiye

**Keywords:** microRNA, cervical cancer, premature ovarian insufficiency, hyperhomocysteinemia, sleep traits, genome, infertility, endometriosis

Reproductive disorders are global health problems affecting millions of people worldwide. In humans, infertility is indeed a very serious disease that disturbs also mental health. In animals, it is not such a big problem, unless it involves, i.e., extinct species or precious specimens for the environment, farming, forestry, etc. In both humans and animals, it reflects abnormalities at many levels of the very complicated fertilization process. The search for the causes of the lack of offspring focuses primarily on the gametes themselves—their fusion is the most important step in the fertilization process. The health of gametes depends on so many factors that it seems improbable that we reproduce.

**FIGURE 1:**
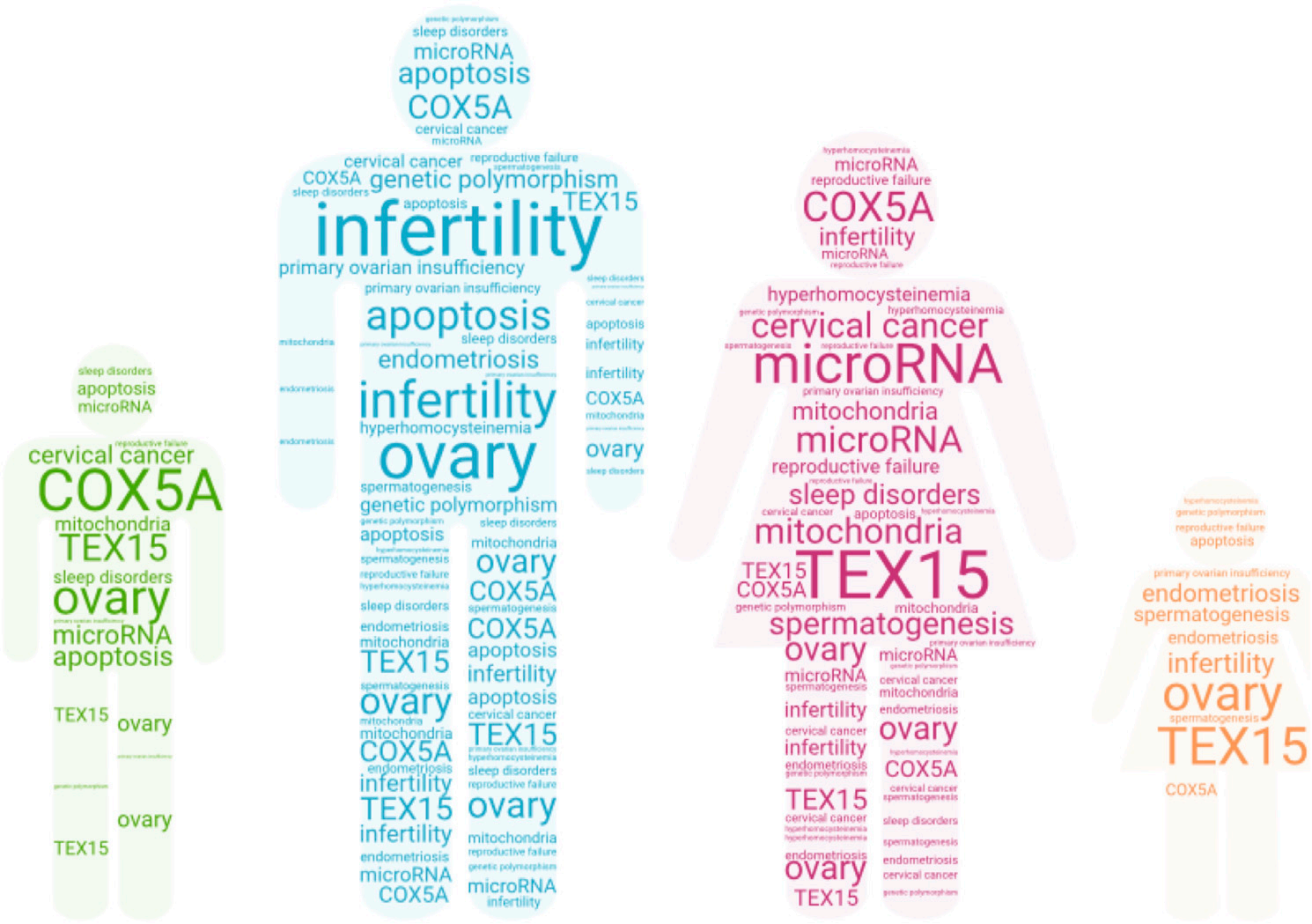
Heath reproductive issues mentioned in the articles’ collection.

The discovery of new pathophysiological mechanisms affecting fertilization is a continuous process forced by the progress of methods. The innovative techniques used, e.g., advanced genomic analysis and functional experiments, provide robust evidence and insights into the genetic basis of reproductive health. Animal models greatly help to study of the fertilization process mechanisms.

The investigation of genetic factors is currently the most promising type of biological research. Here, we recommend a collection of interesting publications on reproductive disorders, cervical cancer and male infertility. We hope that cognition of specific genetic variants and an understanding of the mechanisms involved in reproduction will refine the choice of therapies and contribute to global improvements in human and animal reproductive health.

Three publications in this collection deal with the most serious dysfunctions of the female reproductive tract related to endometriosis, premature expiration of ovarian function, and cervical cancer. Studies of genetic variants indicate new polymorphisms that are candidates as potential biomarkers and could become new therapeutic targets. The publication by Chen et al. concerns genetic polymorphisms in the world’s most common cervical cancer in women. The authors investigated the association between specific genetic variations (SNPs) in microRNA genes involved in the PI3K/Akt signaling pathway and susceptibility to cervical cancer. Numerous researchers have pointed out that these dysregulated microRNAs could play an important role in cervical cancer development (Hillyar et al., 2023; Shen et al., 2020). Some of the stage-specific microRNAs can also be used as biomarkers for cancer classification and monitoring the progression of cervical cancer (Causin et al., 2021). Chen showed that rs107822 of miR-219a and rs2292832 of miR-149 were associated with cervical cancer risk, indicating a potential role of microRNAs in cervical cancer development. They predicted that miR-219a could target integrins (ITGA and ITGB) that participate in the activation of the PI3K/Akt signaling pathway. Thus, the function of these two SNPs in cervical cancer development should be investigated and verified in the future.

In the paper of Wan et al. the association between three *WTAP* (Wilms tumor 1-associated protein) gene polymorphisms and ovarian endometriosis risk in Chinese women was addressed. Results indicated that specific *WTAP* variants may increase susceptibility to ovarian endometriosis among Chinese women. They found that the rs1853259G variant genotypes significantly increased, whereas the rs7766006T variant decreased the association with ovarian endometriosis risk and were correlated with the *WTAP* expression level.

Premature ovarian insufficiency is characterized by early loss of ovarian function before the age of 40 years. This disease has genetic basis. The relationship between novel *CLPP* (caseinolytic mitochondrial matrix peptidase proteolytic subunit) gene variation and premature ovarian insufficiency was investigated by Yuan et al. Results suggested that novel *CLPP* gene variant affects mitochondrial function and triggers granulosa cell apoptosis, potentially contributing to the pathogenesis of premature ovarian insufficiency.

Women’s health is important not only for procreation but also for the health of the offspring Suszynska-Zajczyk et al. investigated the effects of maternal hyperhomocysteinemia induced by a high-methionine diet on the health of their offspring. Elevated homocysteine levels, are a known risk factor for cardiovascular, renal, and neurological diseases as well as pregnancy complications. Results suggested sex-dependent effects on cognition, muscle strength, and breeding outcomes in offspring. A high-metionine diet impairs memory and cognition in female juveniles and weakens muscle strength in male pups. These effects may stem from abnormal placental function affecting early neurogenesis, dysregulation of autophagy-related pathways in the cortex, or epigenetic mechanisms of gene regulation triggered by hyperhomocysteinemia during embryonic development.

Men’s health and the disturbances on the reproductive process are presented the next two publications. Qureshi et al. used whole-exome and genome sequencing to identify novel, clinically significant variants in testis-expressed gene 15 (*TEX15*) in unrelated men with spermatogenic failure (oligozoospermia, nonobstructive azoospermia). The authors found that recessive loss-of-function *TEX15* mutations are associated with spermatogenic failures (meiotic double-strand break repair) in humans and a knockout male mice model.

Lu et al. investigated the association of sleep traits with male fertility, such as the relationship between sleep-related traits (chronotype, sleep duration, insomnia, snoring, dozing, daytime nap, oversleeping, and undersleeping), abnormal sperm or bioavailable testosterone levels using Mendelian randomization analysis. Results suggested a potential link between genetically predicted chronotype and testosterone levels, but no significant association was observed between other sleep traits and male fertility. The authors point out that the human biological clock may be important in reducing the risk of male infertility.

A common Research Topic among all listed studies is the investigation of genetic factors and their impact on various aspects of reproductive health and disease. Using advanced genomic techniques, these studies explored the role of genetic variations (single nucleotide polymorphisms (SNPs) or gene mutations) in conditions such as: cervical cancer, male fertility, spermatogenic failure, premature ovarian insufficiency, endometriosis, and hyperhomocysteinemia-induced deficits in offspring. They also highlighted the importance of understanding the genetic basis of reproductive disorders for improving diagnosis, prognosis, and potential therapeutic interventions. In summary, genetic factors play a significant role in the development and progression of various reproductive disorders, and studying these factors can provide valuable insights into their underlying mechanisms and potential clinical implications (Nagirnaja et al., 2022; Malcher et al., 2022; Turkyilmaz et al., 2022). Despite focusing on different conditions, all studies investigated how genetic variations contribute to disease susceptibility or reproductive health outcomes. Each study contributes valuable insights to its respective field, highlighting the complex interplay among genetics, environmental factors, and disease susceptibility or reproductive health.
